# Obstructive sleep apnea: clinical results of a case treated with an oral appliance

**DOI:** 10.1590/S1808-86942011000400021

**Published:** 2015-10-19

**Authors:** Rafael Golghetto Domingos, José Eduardo Lutaif Dolci, Tomomi Harashima

**Affiliations:** 1 Specialist in orthodontics, CETAO. Researcher of the INPES; 2 Doctoral degree in otorhinolaryngology, São Paulo Federal University. Full professor of otorhinolaryngology, Medical School of the São Paulo Holy House of Mercy Hospital. Member of the Board of Directors of the International Federation of Otorhinolaryngology Societies. Member of the Board of Directors of the Brazilian Otorhinolaryngology Association; 3 Master's degree in endodontic dentistry, Showa University, Tokyo, Japan. Member of the study group on snoring and OSAHS of the Otorhinolaryngology Department, São Paulo

**Keywords:** orthodontic appliances, removable, sleep apnea, obstructive, snoring

## INTRODUCTION

The obstructive sleep apnea hypopnea syndrome (OSAHS) is a respiratory disorder in which collapsed oropharyngeal tissues obstruct the upper airways and interrupt air flow for more than 10 seconds. Apnea means full interruption of air flow in frequent and repetitive events, while hypopnea refers to a 50% decrease in air flow combined with decreased oxyhemoglobin saturation of at least 4%.^1^

The diagnosis of OSAHS may be made based on the clinical and sleep history, which may include Epworth's questionnaire,^2^ which measure the degree of daytime drowsiness; the physical examination and polysomnography - which includes monitoring sleep stages, nasal and oral breathing, oxyhemoglobin saturation, an electrocardiogram, and an electromyography - are added to the diagnostic process.^3^

The number of apnea/hypopnea episodes per hour is named the apnea/hypopnea index (AHI), defined by the American Academy of Sleep Medicine, which classifies OSAHS into mild (AHI from 5 to 15 apnea/hypopnea episodes per hour of sleep), moderate (AHI from 16 to 30), and severe (over 30).^1^

The treatment of OSAHS may be conservative or surgical; several procedures may be indicated, depending on the type of cause of apnea.^4^ These techniques aim to correct the anatomy of patients; the postoperative period is rather uncomfortable, and the results are controversial when assessing the risk/benefit of these procedures - success rates range from 25 to 90%, depending on the type of surgery and the cause of the condition.^4^ Many patients reject surgery, especially the more complex procedures.

A conservative approach that has gained popularity due to its effectiveness in treating sleep respiratory disorders is the use of mandibular repositioning appliances.^5^ These appliances were been accepted by the American Sleep Disorders Association Standards of Practice Committee in 1995; the idea is to increase air flow through the oropharynx by repositioning the mandible downwards and forward.

Among the conditions for using mandibular repositioning appliances, patients should have a minimum amount of teeth in good conditions to keep the appliances in place, and should have no temporomandibular joint issues, which could worsen by using such appliances.^6^

## CASE REPORT

A male white patients (A.C.) aged 33 years, with a BMI of 21.5, and a 12 score in Epworth's questionnaire, presented with snoring, irritability, and daytime drowsiness. The physical examination showed that the general health status was good, the patient's height was 1.75 m, and the weight was 66 kg. An otorhinolaryngological examination revealed:

• normal otoscopy bilaterally;
• left nasal septum deviation, zone 2 (Cottler), and enlarged nasal choanae with mucosa of normal aspect;
• nasofibroscopy: meatuses and choanae with no discharge, positive Müller's maneuver in the oropharynx; larynx with normal aspect;
• mouth and oropharynx: teeth in good state, palate, uvula, and tongue within normal limits, grade I tonsils, Malampati grade II;
• no craniofacial bone alterations. Polysomnography results were: AHI

- 33/h; 2 apneas/h and 31 hypopneas/h; 86% minimum oxyhemoglobin saturation. The dental examination revealed satisfactory dental, periodontal, and temporomandibular conditions. The AHI was classified as severe.

Following the clinical and otorhinolaryngological evaluation, we concluded that the patient had no significant anatomical alteration that could justify surgery, or that could explain the increased AHI; we therefore decided to use a mandibular repositioning appliance.

After the dental clinical examination, molding, placing of the mandibular repositioning appliance, and control, the patient underwent a second polysomnography, which yielded the following results: AHI - 2/h while using the appliance, 0 apneas/h and 2 hypopneas/h, 90% minimum oxyhemoglobin saturation. The BMI in this exam was 21.8.

**Figure 1:**
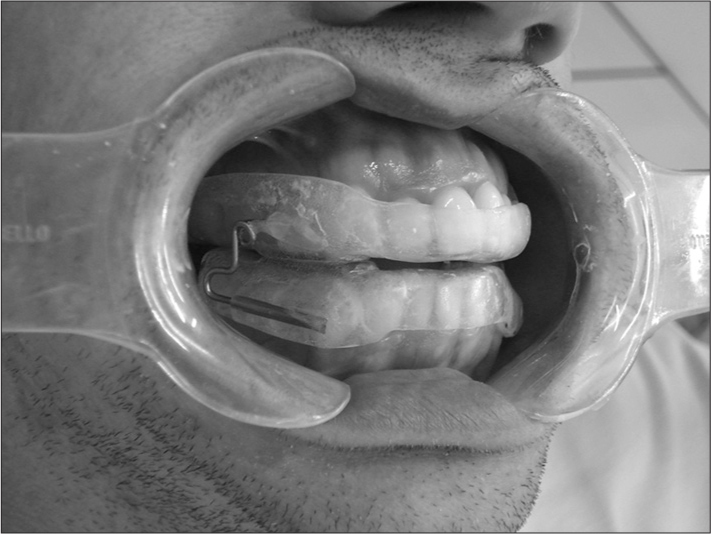
Patient A.C. using the mandibular repositioning appliance.

## DISCUSSION

The treatment of OSAHS should be multidisciplinary because of the multifactor pathophysiology of this condition. The decision for a clinical or surgical approach should be made on an individualized basis. Thus, therapy may start with conservative and less invasive measures, such as mandibular repositioning appliances, speech therapy, nutritional counseling, sleep hygiene, and increasing the awareness and eliciting the cooperation of the patient for the proposed treatment.^6^

## FINAL COMMENTS

Mandibular repositioning appliances effectively reduced the AHI in this patient with OSAHS; it may be used as a conservative approach in place of surgery.
